# The association between problematic short video use and suicidal ideation and self-injurious behaviors: the mediating roles of sleep disturbance and depression

**DOI:** 10.1186/s12889-024-19191-5

**Published:** 2024-06-25

**Authors:** Zhuojun Yu, Xinxin Zhu, Yuanyuan Li

**Affiliations:** 1 Department of Applied Psychology, Guangdong University of Foreign Studies, Guangzhou, China; 2 Department of Psychology, 7 George Square, University of Edinburgh, Edinburgh, EH8 9JZ UK; 3 Department of Psychology, Sun Yat-Sen Memorial Hospital, Sun Yat-Sen University, Guangzhou, China

**Keywords:** Problematic short video use, Sleep disturbance, Depression, Suicide risk, Mediation mechanisms

## Abstract

**Background:**

Prior work suggests that problematic short video use was associated with adverse psychological, physiological, and educational outcomes. With the prevailing of short video platforms, the potential relationships between this problematic behavior and suicidal ideation and self-injurious behaviors have yet to be thoroughly examined. Besides, considering the potential dual nature of problematic short video use, particularly its positive aspects, a potential mechanism may exist linking such problematic behavior to SI and SIBs, ultimately driving individuals towards extreme outcomes. Nevertheless, such mediation paths have not been rigorously examined. Thus, the current study aimed to investigate their relationships and delve into the underlying mechanism, specifically identifying potential mediators between sleep disturbance and depression.

**Methods:**

A quantitative cross-sectional study design was employed to model data derived from a large sample of first- and second-year university students residing in mainland China (N = 1,099; Mage = 19.80 years; 51.7% male).

**Results:**

Results showed that problematic short video use has a dual impact on SI and SIBs. On the one hand, problematic short video use was directly related to the decreased risk of suicidal ideation, attempts, and NSSI. On the other hand, such problematic behavior was indirectly associated with the increased risk of NSSI through sleep disturbance, and it indirectly related to the elevated risk of suicidal ideation, attempts, and NSSI through depression. Besides, on the whole, problematic short video use was positively associated with NSSI but not suicidal ideation and attempts.

**Conclusions:**

These findings indicated that problematic short video use had a dual impact on SI and SIBs. Consequently, it is paramount to comprehend the genuine magnitude of the influence that such problematic behavior holds over these intricate psychological conditions.

**Supplementary Information:**

The online version contains supplementary material available at 10.1186/s12889-024-19191-5.

## Background

With the prevailing of short-video platforms like TikTok, problematic short video use, defined as the addictive use of short-video mediums, has been a worldwide problem among adolescents. Notably, this trend is particularly acute among Chinese first- and second-year university students [27, 44]. This primarily stems from two reasons. First, they may develop a tendency for compensatory indulgence to unleash suppressed energies as they emerge from the stressful high school life. Second, their immature self-control abilities hinder them from making wise decisions in the face of temptation. Such problematic behavior not only leads to severe sleep issues [6, 37], affects emotional stability [27, 42], but also has adverse effects on their academic performance [39]. Even more troubling, in extreme cases, it may trigger suicidal ideation and self-injurious risks (SI and SIBs), posing a significant threat to their physical and mental health. And this idea has been examined on social media platforms [4, 5, 33].

Problematic short video use is akin to the “Band-Aid solution.” On one hand, it may temporarily alleviate stress and provide a brief respite from psychological distress or academic pressures. On the other hand, it may ultimately exacerbate negative emotions and lead to profound, long-term harm as this addictive behavior fails to offer a fundamental solution. However, the potential risks or possibilities of whether problematic short video use would exacerbate or mitigate SI and SIBs have yet to be thoroughly examined. Furthermore, as the next generation of social media, short videos represent a distinct shift from traditional platforms like WeChat. Their unique aspect lies in the fact that they can not only function purely as a medium for browsing to avoid interpersonal communication but also possess a heightened addictive potential due to their yet intense stimuli and profound joy they offer in a short time. Therefore, findings from other social media applications or the Internet may not readily apply to short video applications.

Why does problematic short video use ultimately increase the risk of SI and SIBs? Past studies have not adequately answered this question. Considering the dual nature of problematic short video use, particularly its positive aspects, a potential mechanism may exist linking such problematic behavior to SI and SIBs, ultimately driving individuals towards extreme outcomes. Existing research demonstrated the association between problematic short video use and sleep disturbance [6, 37], as well as the subsequent link between sleep disturbance and SI and SIBs [8],J.-W. [12, 20], bolstering the argument for the potential mediating role of sleep disturbance in this relationship. Besides, empirical evidence has found that problematic short video use was correlated to depression [27, 42], and previous meta-analyses show that depression is a significant risk factor for SI and SIBs [28, 38], providing support for the potential mediating role of depression. Nevertheless, such mediation paths have not been rigorously examined.

In all, previous studies are limited in examining the relationship between problematic short video use and SI and SIBs, particularly its positive effect. Additionally, there are no studies directly evaluate the simple and serial mediating effect of sleep disturbance and depression in their relationships.

To fill the research gaps, adopting a cross-sectional design, this study aims to examine the relationships between problematic short video use and suicidal ideation, attempts, and NSSI. Additionally, by identifying the potential mediators of sleep disturbance and depression, our study may help to identify the most appropriate intervention and prevention targets for reducing adolescents’ suicide risk affected by problematic short video use.

**Fig. 1:**
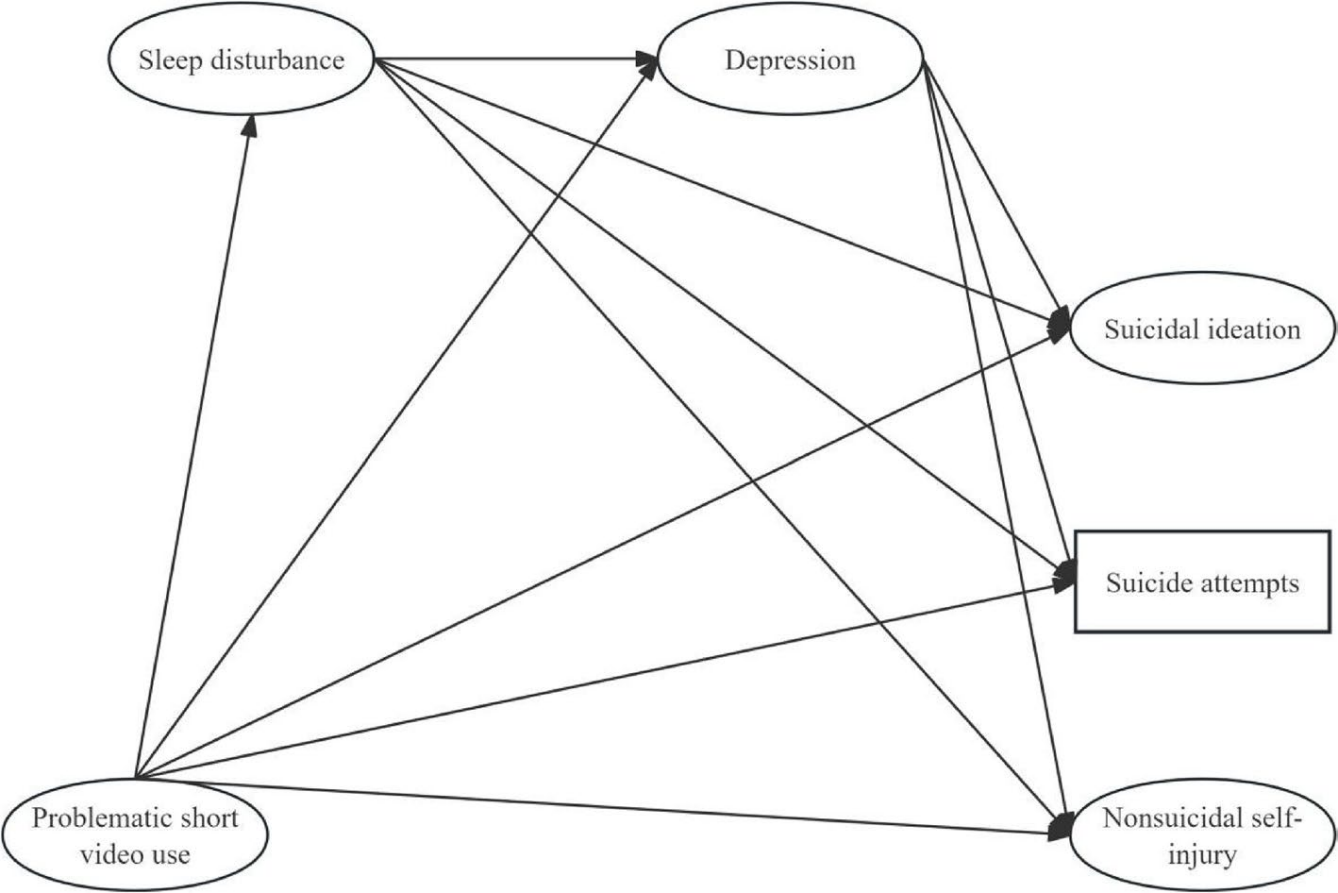
Theoretical model

In the following section, we discuss the detail of our theoretical model using Fig. 1 as a guide.

### The negative and direct association between problematic short video use and suicidal ideation and self-injurious risks

Initially, short video use provides a sense of instant relaxation and happiness. Such problematic behavior may effectively mitigate the risk of suicide and self-harm among adolescents. Just like other forms of social media, a limited number of studies have reported that social media use or addiction was positively associated with SI and SIBs. For example, a meta-analysis suggested that some social media/internet use, compared to no use, may be associated with fewer suicide attempts among young people less than 19 years old [29]. Besides, a cross-sectional study surveyed 40,065 Norwegian college and university students (age range 18–25 years) and found that passive non-social use of social media was associated with decreased odds of NSSI ideation, NSSI, and suicidal ideation, and active non-social use was associated with reduced odds of suicide attempt [13]. Furthermore, one study recruited 374 university students (Mage = 20.01, SD = 1.84) and showed a negative cross-sectional relationship between social media addiction and recent two-week suicidal ideation when depression was taken into account [24]. Therefore, problematic short video use, as the next generation of social media/Internet platforms, may share a similar impact on suicidal ideation and self-injurious behaviors as problematic social media use. This problematic behavior may offer a timely respite from stress in the beginning, serving as a protective buffer against SI and SIBs. Accordingly, Accordingly, our first hypothesis predicted that problematic short video use will be directly associated with decreased risks of suicidal ideation, attempts, and NSSI.

### The potential mediating role of sleep disturbance

Although problematic short video use can relieve momentary stress, its effectiveness is analogous to the “Band-Aid solution.” Overreliance on such videos, in the long run, not only fails to address the underlying challenges but can foster further issues, such as sleep disturbance, ultimately escalating the risk of SI and SIBs.

Problematic short video use typically manifests as an inability for individuals to resist the urge to immerse themselves excessively, a stark reflection of a severely compromised self-control ability. This problematic behavior can significantly disrupt an individual's natural circadian rhythm, leading to an uncontrollable sleep–wake pattern that ultimately culminates in sleep disturbance [6, 37]. Several studies have suggested a positive association between problematic short video use and sleep problems. For instance, one cross-sectional study, employing Chinese adolescents (*n* = 1,346, Mage = 14.97, SD = 1.13), suggested that individuals addicted to short video use exhibited lower sleep quality than nonusers and moderate users [6]. Another study, with a sample of 1,050 TikTok/Douyin users in China, focused on how TikTok use may interfere with individuals’ circadian rhythms and showed that automatic TikTok use might render individuals to experience higher levels of cognitive arousal before sleep, which may be further associated with increased daytime fatigue [37].

Furthermore, systematic reviews and meta-analyses demonstrated that adolescents and young adults with sleep disturbance were at higher risk of suicidal ideation, attempts, and NSSI than those without sleep complaints [8],J.-W. [12, 20]. There are small-to-medium to medium effect sizes for sleep disturbance and suicidal ideation, attempts, and death (see [21], for meta-analysis). In examining the effects of specific sleep disturbance, another meta-analysis of longitudinal studies suggested that the strongest effects were found for insomnia, which significantly predicted suicide ideation, and nightmares, which significantly predicted suicide attempts [10].

Therefore, sleep disturbance may be a significant potential mediator in the association of problematic short video use with suicidal ideation and self-injurious behaviors. Thus, our second hypothesis predicted that problematic short video use will be indirectly associated with higher risks of suicidal ideation, attempts, and NSSI through sleep disturbance.

### The potential mediating role of depression

In addition to a decline in the ability to manage sleep–wake cycles, problematic short video use may also weaken an individual’s ability to regulate their emotions, thereby intensifying the risk of depression [27, 42]. Existing literature has reported that problematic short video use was positively associated with depression. For example, a study, using a cross-lagged panel network analysis, indicated that specific symptoms of short video addiction, namely “Tolerance,” and depressive symptoms such as “Anhedonia,” predicted the subsequent development of symptoms for each mental health issue. Besides, both the addiction problem “Conflict” and emotional feelings such as “Sad mood” may serve as bridge symptoms linking the co-occurrence of these two mental health issues in Chinese college students (*n* = 1163, Mage = 19.46, SD = 1.04) [27]. Furthermore, a cross-sectional study recruited 400 Pakistani university students (age range 18–36 years,63% of them were studying at the bachelor's level and found an association between TikTok addiction and depression and anxiety [42].

Given that depression serves as a proximal factor for suicide, problematic short video use may exacerbate negative emotions and stress for vulnerable individuals, ultimately prompting them to consider suicide as a means to escape extreme distress, thereby fostering suicidal ideation and potentially leading to the development of suicidal behaviors as an increasingly radical coping strategy. Therefore, depression may play a mediating role in the association between problematic short video use and SI and SIBs. Thus, our third hypothesis predicted that problematic short video use will be indirectly associated with higher risks of suicidal ideation, attempts, and NSSI through depression.

Theoretically, according to social zeitgeber theory [7], problematic short video use (as a life event) can increase the risk of depressive episodes by disrupting individuals’ social routines and, hence, their biological circadian rhythms. In addition, social zeitgeber theory has been suggested as potentially applicable to suicide [3],however, the idea has yet to be tested. Therefore, our fourth hypothesis predicted that problematic short video use would be indirectly associated with higher risks of suicidal ideation, attempts, and NSSI through depression through both sleep disturbance and depression.

### The positive and overall association between problematic short video use and suicidal ideation and self-injurious risks

Ultimately, problematic short video use may trigger a cascade of physical and psychological issues, increasing the risk of SI and SIBs. Previous studies have demonstrated that problematic short video use can yield various adverse outcomes among adolescents. For instance, in terms of psychological outcomes, a study surveyed 400 Pakistani university students (age range 18–36 years; 63% of them were studying at the bachelors level) and found an association between TikTok addiction and depression and anxiety [42]. Regarding physiological outcomes, one study, recruiting Chinese senior high school students (*n* = 3,036, Mage = 16.56, SD = 0.62), indicated that TikTok use disorder is positively linked to memory loss [30]. In terms of educational outcomes, a cross-sectional study indicated problematic short video use not only directly impacted academic procrastination but also had an indirect effect on academic among Chinese college students (*n* = 1,176, Mage = 20.08, SD = 1.34) [39].

Furthermore, like other social media/Internet forms, previous studies have demonstrated the positive relationships between problematic social media/Internet use and SI and SIBs. Specifically, a meta-analysis and a systematized narrative review suggested that problematic social media use was cross-sectionally associated with suicidal ideation among adolescents and adults, with a small to medium effect [4, 26]. Besides, a longitudinal study indicated that problematic Facebook use was positively related to one-year follow-up suicidal ideation and attempts among German adults (*N* = 209, Mage = 23.01, SD = 4.45) [5]. Additionally, a multicenter and cross-sectional survey study, using a large Chinese sample (*N* = 15, 623, age range 11–20 years), showed that both possible internet addiction and internet addiction were associated with less-frequent or more-frequent Non-suicidal self-injury (NSSI) [33]. Accordingly, our fifth hypothesis predicted that problematic short video use will be totally associated with higher risks of suicidal ideation, attempts, and NSSI.

## Method

### Participants & Procedures

The cross-sectional study was conducted from 7 Dec (2023) to 10 Dec (2023). We recruited participants through a link published on a China-based social media platform (WeChat). With the informed consent of all participants, the authors confirmed their identity information before the survey. Participants were eligible for the study and invited to participate if they: (a) were first-or second-year students (during late adolescence and emerging adulthood), (b) agreed to participate in the study, and (c) were under closed school management during the COVID-19 pandemic. Across an assessment, the participants received identical written instructions from the questionnaire. They were allowed to take as much time as needed to complete the measures questionnaire and withdraw from the study at any time.

In total, 1,209 participants were invited to this study. We excluded 200 people from our analyses because of incomplete responses. The final study population included 1,009 participants (Mage = 19.80 years, approximately 51.7% male, 24.2% Freshman).

### Ethics

This study is in agreement with the Helsinki Declaration and Chinese national guidelines for ethics in research (“Criterions for the Quality Control of Clinical Trial of Drugs” as well as the “Management Measures for Researcher-Initiated Clinical Research in Medical and Health Institutions” (Trial Implementation)). The study protocol was approved by the Medical Ethics Committee of Sun Yat-sen Memorial Hospital (SYSKY-2023–1200-01). All the participants were assured of their responses' confidentiality and could withdraw from the survey at any time without any reason.

### Measures

#### Problematic short video use

Problematic short video use was defined as the addictive use of such mediums encompassing short-video applications, websites, micro-programs, and the like. The manifestation of this addictive behavior closely aligns with the defining characteristics of addiction outlined in the Bergen Social Media Addiction Scale (BSMAS; [1]). The BSMAS is arguably the most well-established problematic use scale, and these items capture the six core criteria of behavioral addiction, namely salience, mood change, tolerance, withdrawal, conflict, and relapse [1]. In the absence of a pre-existing scale to measure problematic short video use, we adapted the Chinese version of the BSMAS (BSMAS-CV; [18]) to fit the context of short videos. The BSMAS-CV consists of 6 items based on the six core components. Each item was answered on a 5-point Likert scale, ranging from 1 (very rarely) to 5 (very often).

In the process of adaptation, the word “social media” was replaced with “short video” (e.g., “How often during the last year have you spent a lot of time thinking about short video or planned use of short video?”). Moreover, we added a description about short videos, indicating that short video use refers to individuals subscribing, commenting on, and creating videos with short video applications (e.g., Douyin, Kuaishou, Mepai, pear video) and social media with video-sharing capabilities (e.g., WeChat, Xiaohongshu).

A mean score was analyzed, and higher scores indicated higher problematic short video use levels. Psychometric properties of the BSMAS-CV have been supported among young adults from Hong Kong and Taiwan (24.08 years ± 5.06 vs. 20.51 years ± 1.22) [18]. Its Cronbach α was 0.883.

#### Suicidal ideation

Suicidal ideation was measured by the Chinese version of screening items of the Beck Scale for Suicide Ideation (BSI-CV-Screen; [2, 19]). The BSI-CV scale comprises two subscales: suicide ideation (Items 1–5) and suicidal intent (Items 6–19). Specifically, Items 1–5 are tailored for screening suicide ideation, while Items 6–19 are frequently utilized to assess the severity of such ideation (e.g., [14, 19]). Given that our target population comprises regular college students, not individuals already exhibiting suicidal thoughts, and considering the sensitivity of suicide ideation in China, we have opted to utilize the first five items exclusively for screening purposes. This approach ensures we can identify potential risks without imposing undue stress on our participants.

In this scale, participants reported their suicidal thoughts during the past 12 months. Response options reflected a 3-point Likert scale ranging from 0 (*not at all*) to 2 (*extremely*). A mean score was computed, with higher scores indicating higher risks of suicidal ideation. Psychometric properties of the BSI have been supported in the Chinese version among adolescents (age range 17–21 years) and university students [19, 43]. Its Cronbach α was 0.833.

#### NSSI

NSSI was measured using the first section of the Inventory of Statements About Self-Injury (ISAS; Chinese version translated by two Chinese Ph.D. students in psychology with a good bilingual background) [15, 16]. The translation process included three steps. The original English version was translated first into Chinese translations by one student. And then, another student translated the questionnaire back into English. Finally, to adapt the translated version to the Chinese culture, they checked the final translation and corrected any remaining spelling, diacritical, grammatical, or other errors.

In this section of the ISAS, twelve different NSSI behaviors performed “intentionally (i.e., on purpose) and without suicidal intent” will be assessed, including banging/hitting self, biting, burning, carving, cutting, wound picking, needle-sticking, pinching, hair pulling, rubbing skin against rough surfaces, severe scratching, and swallowing chemicals. Participants were asked about the frequency of each NSSI behavior they had performed during the past 12 months (e.g., banging or hitting self). This scale is rated on a 6-point scale, ranging from 0 (*never*) to 5 (*5 or more times*). A mean score was analyzed, with higher scores indicating higher NSSI frequencies. Its Cronbach α was 0.959 (see Tables 1 and 2 for the ISAS and the Chinese version of ISAS).

**Table 1:** The Chinese version of Inventory of Statements About Self-Injury (ISAS-CV).在过去一年中, 你是否有以下故意伤害自己的行为 (但没有任何自杀或者结束自己的生命的意图或倾向) ?请圈出最符合你个人情况的数字 (每题只能选1个)

	0次	1次	2次	3次	4次
(1)故意猛烈碰撞自己 (如头部或其他身体部位) 以致瘀伤					
(2)故意咬伤自己					
(3)故意烧伤自己					
(4)故意在皮肤上刻字或画图以致出血或留疤痕					
(5)故意割伤自己					
(6)故意抓挠伤处或弄伤已经开始痊愈的伤口					
(7)故意将尖锐的物体, 如针、钢钉、订书针等插入皮肤或指甲					
(8)故意掐 (或拧) 自己					
(9)故意使劲儿拽头发					
(10)故意在粗糙的物体表面上猛烈摩擦皮肤					
(11)故意严重抓伤自己以致出血或留疤痕					
(12)故意服用有毒性化学药物

**Table 2:** The Inventory of Statements About Self-Injury (ISAS). In the past 12 months, have you engaged in any of the following self-injurious behaviors (without any intention or tendency to commit suicide or end your life)? Please circle the number that best reflects your personal situation (only one option per question)

	0 times	1 times	2 times	3 times	4 times
(1)Banging or hitting self					
(2)Biting					
(3)Burning					
(4)Carving					
(5)Cutting					
(6)Wound picking					
(7)Needle sticking					
(8)Pinching					
(9)Hair pulling					
(10)Rubbing skin against rough surfaces					
(11)Severe scratching					
(12)Swallowing chemicals					

#### Suicide attempts

Suicide attempts were measured with one item: “During the past 12 months, how many times did you actually attempt suicide.” The item came from the Youth Risk Behavior Survey, a cross-sectional study focusing on adolescent health risk behaviors [40]. Responses were given in 5-point format with response options of 0 = *0 time*, 1 = *1 time*, 2 = *2 or 3 times*, 3 = *3 or 4 times*, 4 = *4 or 5 times*, and 6 = *6 or more times*. Higher scores indicate higher risks of suicide attempts.

#### Sleep disturbance

Sleep disturbance was measured using the 8-item Patient-Reported Outcomes Measurement Information System Sleep Disturbance Short Form (Chinese version translated by two Chinese students in psychology) [41]. The translation procedure followed three steps. Firstly, one researcher with postgraduate qualifications translated the English version into simplified Chinese in the forward translation. Secondly, back translation was performed by one other Ph.D. in psychology. She has a good bilingual background and had not been involved in the forward translations, translated the Chinese version into English. Finally, they checked the simplified Chinese and back-translated English versions to assess the semantic equivalence.

In this scale, participants rated three aspects (i.e., sleep satisfaction, quality, and disturbance) of their sleep over the past seven days on a 5-point scale. Most of the items used an intensity scale ranging from 1 (*not at all*), 2 (*a little bit*), 3 (*somewhat*), 4 (*quite a bit*), to 5 (*very much*), with a smaller number using a frequency scale ranging from 1 (*never*), 2 (*rarely*), 3 (*sometimes*), 4 (*often*), to 5 (*always*); and one item assessing overall sleep quality using a scale ranging from 1 (*very poor*), 2 (*poor*), 3 (*fair*), 4 (*good*), to 5 (*very good*). Items 2, 3, 7, and 8 were reverse-scored. A mean score was analyzed, with higher scores indicating higher levels of sleep disturbance. Its Cronbach α was 0.861.

#### Depression

Depression was measured using the 21-item Depression Anxiety Stress Scale 21 (DASS-21; Chinese version; [22, 34]). More specifically, it assesses depression with seven items (i.e., items 3, 5, 10, 13, 16, 17, 21). All items are rated on a four-point Likert scale ranging from 0 (*did not apply to me at all*) to 3 (*applied to me very much*). The higher mean score indicates a higher level of depressive symptoms. Psychometric properties of the DASS-21 have been supported in the Chinese version among emerging adults (Mage = 19.00 years) [9]. Its Cronbach α was 0.918.

### Data analysis

The hypothesized mediation model was evaluated using Mplus 7.4 [25] with the the Maximum Likelihood (ML) estimation. Specifically, to estimate the relationships between problematic short video use and SI and SIBs, and the mediators of sleep disturbance and depression, we examined all the paths from problematic short video use to suicidal ideation, attemtps, and NSSI in the model. Considering the measures of our study (all the factors are manifest variables), we did not report the model fit information. We tested the significance of the indirect effect using bootstrapped estimates (based on 10,000 bootstrapped samples, [35]), which provides percentile confidence intervals [23]. The indirect effect was statistical significance if the 95% confidence interval (CI) did not include zero [31].

## Results

### Descriptive statistics

The means, standard deviations, and bivariate correlations of problematic short video use, sleep disturbance, depression, and suicidal ideation/attempts/NSSI are presented in Table 3. Almost all variables were positively correlated with each other except for the correlation between problematic short video use and suicidal ideation.

**Table 3:** Descriptive statistics and correlations among study variables (*N* = 1, 009)

	Mean	SD	PSVU	SD	D	SI	SA	NSSI
PSVU	3.22	.82	1					
SD	2.10	.66	.15***	1				
D	1.65	.68	.29***	.62***	1			
SI	1.18	.34	.05	.37***	.53***	1		
SA	1.26	.66	.07*	.29***	.46***	.59***	1	
NSSI	1.35	.77	.14***	.36***	.51***	.51***	.58***	1

*PSVU* Problematic short video use, *SD* Sleep disturbance, *D* Depression, *SI* Suicidal ideation, *SA* Suicide attempts, *NSSI* Non-suicidal self-injury

^*^ *p* < .05. **p < .01. ***p < .001

### Testing the Mediation Model

**Fig. 2:**
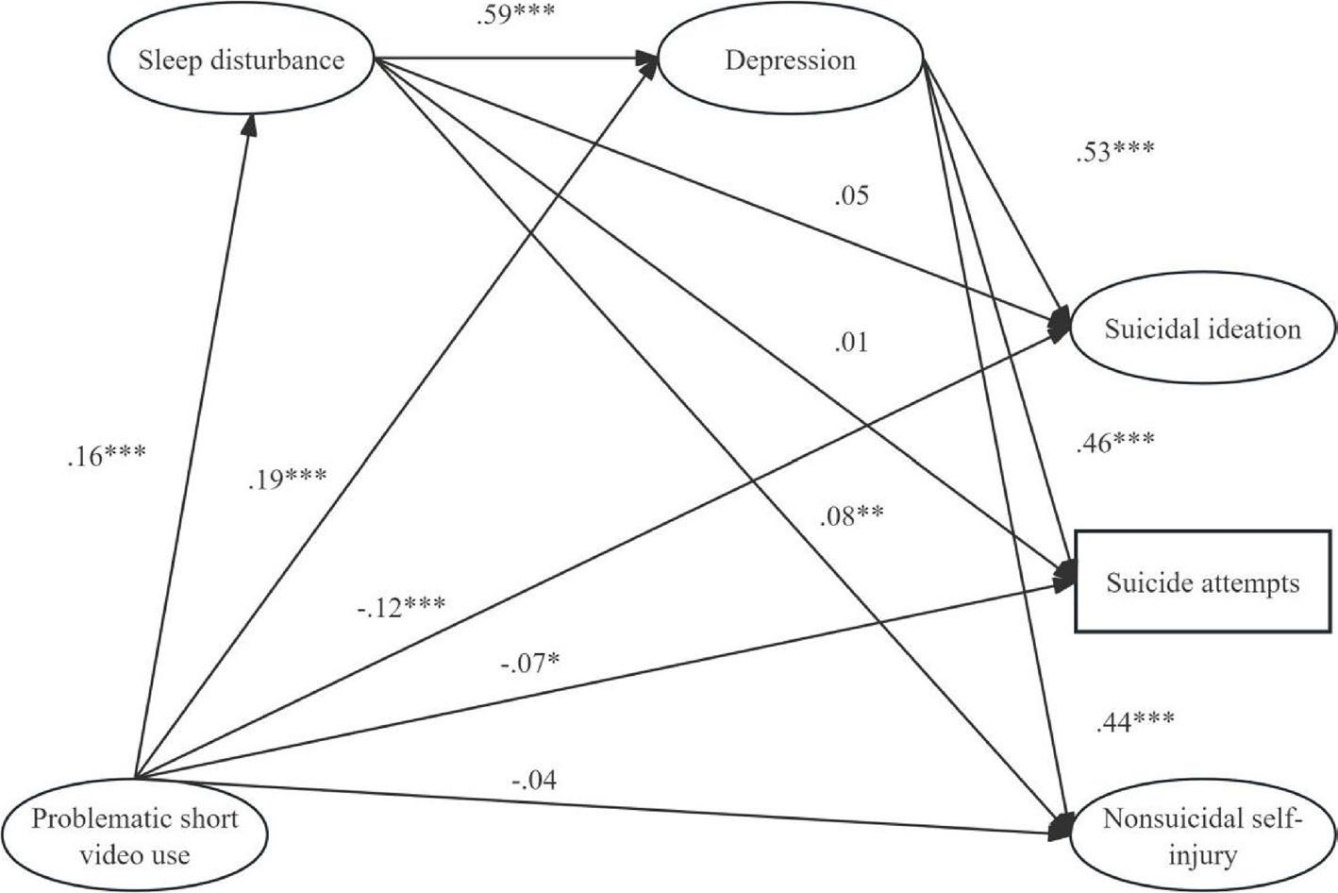
Mediating effects of sleep disturbance and depression on the association between problematic short video use and suicidal ideation, attempts and NSSI (*N* = 1,009). *p < 0.05; **p < 0.01; ***p < 0.001

Tables 4, 5, and Fig. 2 showed the main results with covariates included in our mediation model. Results, presented in Table 5, showed that problematic short video use was totally and positively associated with adolescents’ NSSI, but the total effect of problematic short video use on suicidal ideation and attempts was not significant.

**Table 4:** Model-based effect sizes for the observed correlations (*N* = 1, 009)

Relationship		β	Effect Size (*r*)	95% CI
Var.1	Var.2			
PSVU	D	.19	.69	[.14, .24]
SD	D	.59	1.09	[.55, .63]
PSVU	SD	.16	.66	[.09, .23]
PSVU	SI	-.12	-.12	[-.17, -.06]
SD	SI	.05	.55	[-.02, .11]
D	SI	.53	1.03	[.45, .61]
PSVU	SA	-.07	-0.07	[-.13, -.02]
SD	SA	.01	.51	[-.06, .07]
D	SA	.46	.96	[.38, .55]
PSVU	NSSI	-.04	-.04	[-.09, .01]
SD	NSSI	.08	.58	[.02, .14]
D	NSSI	.44	.94	[.37, .52]

*PSVU* Problematic short video use, *SD* Sleep disturbance, *D* Depression, *SI* Suicidal ideation, *SA* Suicide attempts, *NSSI* Non-suicidal self-injury

*r* = *β* + 0.5*λ* (*λ* is an indicator variable that equals 1when *β* is nonnegative and 0 when *β* is negative)

**Table 5:** Mediating effects with covariates for problematic short video use on suicidal ideation and self-injurious behaviors (*N* = 1,009)

Total, direct and indirect paths	β	95% CI	∣a * b / c’∣ (%)
Total			
PSVU → SI	.04	[-.02, .10]	
PSVU → SA	.06	[.00, .12]	
PSVU → NSSI	.10	[.04, .16]	
Direct			
PSVU → SI	-.12	[-.17, -.06]	
PSVU → SA	-.07	[-.13, -.02]	
PSVU → NSSI	-.04	[-.09, .01]	
Indirect			
PSVU → SD → SI	.01	[-.003, .02]	
PSVU → SD → SA	.001	[-.01, .01]	
PSVU → SD → NSSI	.01	[.003, .03]	34.21%
PSVU → D → SI	.10	[.07, .13]	86.32%
PSVU → D → SA	.09	[.06, .11]	123.94%
PSVU → D → NSSI	.08	[.06, .11]	221.05%
PSVU → SD → D → SI	.05	[.03, .07]	42.74%
PSVU → SD → D → SA	.04	[.03, .06]	61.97%
PSVU → SD → D → NSSI	.04	[.02, .06]	110.53%

*PSVU* Problematic short video use, *SD* Sleep disturbance, *D* Depression, *SI* Suicidal ideation, *SA* Suicide attempts, *NSSI* Non-suicidal self-injury

Results of component paths, presented in Table 4 and Fig. 2, showed that (1) problematic short video use was positively associated with both sleep disturbance and depression, negatively associated with suicidal ideation and attempts, but not associated with NSSI; (2) sleep disturbance was positively related to depression and NSSI, but not related to suicidal ideation and attempts. (3) depression was positively associated with suicidal ideation, attempts, and NSSI.

We then used the bootstrapping procedure to test these significant indirect paths observed by path analysis. Results indicated that (1) problematic short video use was directly and negatively associated with suicidal ideation, attempts, and NSSI; (2) sleep disturbance mediated the association between problematic short video use and NSSI; (3) depression mediated the association between problematic short video use and suicidal ideation, attempts, and NSSI; (4) serial mediating effects in the association between problematic short video use and suicidal ideation, attempts, and NSSI by sleep disturbance and depression were significant.

### Additional analyses

Additional analyses were performed to provide more details on these associations. First, as we employed a structural equation model (SEM) to conduct a robust analysis, we have included a supplementary report on the reliability of the three sub-dimensions associated with sleep disturbance. Specifically, sleep satisfaction was measured using items 1, 2, 3, and 4. Its Cronbach α was 0.722. Sleep disturbance was measured using items 5, 6, and 7. Its Cronbach α was 0.710. Sleep quality was measured using item 8.

Second, the SEM was used to analyze the relationships between problematic short video use and SI and SIBs and the mediating role of sleep disturbance and depression. The model fit indices of the SEM were all within specifications (χ2/df = 4.34, *P*-value < 0.00, CFI = 0.92, TLI = 0.91, RMSEA = 0.06, and SRMR = 0.05), indicating good model fit. The SEM results are consistent with path analysis except for the non-significant mediating effect in the relationship between problematic short video and NSSI. However, the 95% CI of this mediating effect is close to 0 in both the SEM and path analysis results (see Table S1- S2 and Fig. S1 in the supplementary materials).

Third, we conducted sensitivity analyses using regression diagnosis to exclude influential cases or outliers and help assess the robustness of the observed correlations. The sensitivity analyses show that except for the inconsistent and non-significant coefficient of sleep disturbance in the model M25 of Table S3-S4, all the coefficients of variables are consistent with the results run on the original sample (see Table S3-S4 in the supplementary materials).

## Discussion

### The dual impact of problematic short video use on suicidal ideation and suicde attempts

Consistent with our hypothesis, problematic short video use can trigger significant psychological problems. However, such problematic behavior possesses a dual nature.

Our findings on the direct effect of problematic short video use indicated that it may provide a temporary respite, acting as a buffer against self-injury (SI) and suicidal ideation and behaviors (SIBs). This temporary relief seems to stem from three primary factors. Firstly, adolescents may find emotional support and understanding in the optimistic content of short videos, thereby alleviating psychological stress. Secondly, these videos may facilitate social interaction and connectivity among adolescents, providing them with social support to address practical problems and mitigate extreme outcomes. Lastly, a strong desire and dependency on short videos can temporarily distract from real-life difficulties, reducing the risk of SI and SIBs. However, it is crucial to emphasize that this relief is temporary and does not address the underlying causes of the problem.

Just as the “Band-Aid solution,” our findings found that the indirect effect manifested as a masking effect, which was the opposite of the direct impact. Specifically, problematic short video use was positively related to SI and SIBs via the simple mediating role of depression or the serial mediating roles of sleep disturbance and depression. Therefore, while problematic short video use may offer temporary stress relief, in the long run, this addictive behavior can lead to a range of negative physiological and psychological consequences, ultimately heightening the risk of SI and SIBs.

Considering the dual nature of problematic short video use, we have underestimated the overall impact of problematic short video use on suicidal ideation, attempts, and NSSI. This oversight is significant as it fails to fully capture the true extent of the influence of such problematic behavior on these complex psychological conditions. Thus, the lack of significant correlations between problematic short video use and suicidal ideation and attempts is comprehensible. Moreover, given that the sample we recruited primarily comprised ordinary college students, the relatively small number of individuals exhibiting suicidal or self-injurious tendencies may also have contributed to the non-significant results observed. Therefore, future research could concentrate on vulnerable adolescent populations, specifically those with mental health concerns or a history of suicidal or self-injurious thoughts or behaviors, which may provide more insight into the relationships between problematic short video use and SI and SIBs. Nonetheless, our finding showed the significant total effect of problematic short video use and NSSI, which was consistent with the findings on social media (see [26], for meta-analysis).

### The mediating role of depression

Upon examining the potential mechanism between problematic short video use and SI and SIBs, our study identified depression as an important mediating variable. First of all, our findings similarly underscore the significance of depression as a crucial predictor of suicidal ideation, suicidal attempts, and non-suicidal self-injury (NSSI) among adolescents, which is consistent with prior studies [28, 38], for meta-analyses).

Besides, we also found a significant positive correlation between problematic short video use and depression, also in accord with existing research [27, 42]. Notably, given that depression is an extremely negative emotion like SI and SIBs, theoretically, there should also exist two distinct effects between such problematic behavior and depression. However, our findings solely demonstrate the positive effect of problematic short video use on depression, lacking the potential masking effect. A possible explanation is that the gratification derived from this addictive behavior tends to be fleeting and superficial and can only serve as a temporary respite for impulsive or immediate emotional fluctuations such as SI and SIBs. Thus, it is unable to reverse the effect of depression, as it is a deepening and relatively stable emotional state.

### The mediating role of sleep disturbance

Just as problematic short video use can exacerbate the risk of depression, our research similarly indicated that such behavior can also intensify sleep disturbance among adolescents. The reason may lie in the fact that problematic short video use excites and captivates them, making it difficult for them to enter a state of restful sleep. Based on this observation, problematic short video use is unlikely to alleviate sleep disturbances.

Moreover, our research findings suggested that there is no significant direct correlation between sleep disturbance and suicidal ideation or attempts. Even though there is a positive mediating effect in the relationship between problematic short video use and NSSI, the 95% CI of this mediating effect was close to 0. However, there is a marked association with psychological issues such as depression, suggesting that while sleep disturbance alone does not trigger extreme behaviors, it can indeed precipitate mental illnesses like depression and exacerbate their symptoms.

Hence, our findings revealed that while sleep disturbances do not serve as a simple mediating factor between problematic short video use and suicidal ideation or attempts, they exhibited a minor mediating role in the relationship between such problematic behavior and NSSI. However, when viewed through the lens of a longer chain of mediation, problematic short video use via sleep disturbances and depression acts as a significant amplifier, considerably increasing the risks of suicidal ideation, attempts, and NSSI.

It indicates that sleep disturbance is a crucial generative mechanism variable. That is when sleep disturbance is significantly correlated with negative psychological issues that are linked to suicide, it can heighten the likelihood of SI and SIBs among adolescents who are already addicted to short videos. These findings are consistent with the social zeitgeber theory [7] and the three-step theory [17]. According to these two theories, problematic short video use may disrupt adolescent circadian rhythms of sleep and thus reduce the ability to regulate emotions, leading to an increase in symptoms of depression over time. If depression (as a pain) can not be diminished with time or effort, adolescents may develop suicidal ideation and further progress to action.

### The comparison with other research in different cultural backgrounds

It is noteworthy that previous studies investigating the relationships between social media/internet addiction and SI and SIBs have yielded inconsistent findings across different cultural contexts. For example, a cross-sectional study surveyed 40,065 Norwegian college and university students (age range 18–25 years) and found that passive non-social use of social media was associated with decreased odds of NSSI ideation, NSSI, and suicidal ideation, and active non-social use was associated with reduced odds of suicide attempt [13]. However, a longitudinal study indicated that problematic Facebook use was positively related to one-year follow-up suicidal ideation and attempts among German adults (*N* = 209, Mage = 23.01, SD = 4.45) [5].

However, we observed that despite Norway and Germany sharing similar cultural backgrounds, they arrived at different conclusions, while China and Germany reached similar conclusions despite their diverse cultural landscapes. This may suggest that the relationships between social media/internet addiction and SI and SIBs may not be strongly correlated with cultural background. Instead, the dual-faceted impact of problematic short video use pointed out in this study could offer a more plausible explanation. Consequently, future research could delve deeper into the dual relationships between problematic short video use and SI and SIBs across various national samples, thereby providing a more comprehensive understanding of this complex phenomenon.

Nevertheless, we can not ignore the significance of cultural backgrounds. There are indeed studies that underscore the importance of cultural factors as a critical moderating variable, potentially influencing the strength of the relationship between problematic short video use and SI and SIBs. In collectivist societies, previous research has indicated that cultural elements with a high degree of constraint may mitigate the negative impact of social media usage on mental health. For example, a cross-sectional study, with a sample of 391 young Jordanian adults, demonstrated that the greater the cultural restraint, the less social media motivations influence subjective happiness [11]. Another cross-sectional study recruited 709 college students, indicating that religion mitigated the relationship between social media addiction and mental problems [32]. Although we did not find a moderating effect of cultural factors associated with individualistic societies in the relationships between social media/Internet addiction and SI and SIBs in existing studies, cross-national research revealed that American students who prioritize personal entertainment satisfaction exhibit different trajectories of problematic short video use than Chinese students who focus on social entertainment needs [44]. In summary, cultural factors may either enhance or attenuate the strength of the relationships between problematic short video use and its association with SI and SIBs. Future research is also needed to delve deeper into exploring whether the duality of the effects of such addictive behavior manifests differently in varying cultural contexts or operates through varying mechanisms.

### Limitations and Future directions

There were some limitations of this study bear noting. First, our study encompassed a sample of Chinese first- and second-year university students. This demographic offers a glimpse into the Chinese late adolescent population aged 17–24. However, the generalizability of our findings to a broader demographic remains a matter for further exploration. Second, given that our sample was primarily collected during the COVID-19 lockdown period when schools implemented strict management measures, it is plausible that there was an increase in problematic short video use among adolescents. This augmentation in usage presents a unique opportunity to observe the correlations between problematic short video use and SI and SIBs. However, it is essential to acknowledge that this sample collection method may overestimate the strength of the relationships between problematic short video use and SI and SIB. Third, all variables in this study were measured using self-report questionnaires, which might be subject to recall bias or social desirability bias. Fourth, due to this study's space limitation, we have primarily focused on exploring the underlying mechanisms of influence without incorporating conditional mechanisms into our analysis.

Hence, future research could further delve into the topics pertinent to this study through the following approaches. First, employing a longitudinal research design incorporating multiple time points can help identify more reliable causal relationships. Second, expanding the study’s scope from late adolescents to adolescents spanning the entire age range of 15 to 24 yearsold (an age group referred to as “youth” by the United Nations; UN, [36]) and conducting the research in a relatively controlled and stable environment would be instrumental in validating the generalizability of our findings. Third, employing research methods such as survey experiments, which significantly reduce social biases, can greatly aid in testing the robustness of our study's findings more precisely. Lastly, future research could incorporate moderators such as social support, coping strategies, and personality traits into its study design to more comprehensively explore the dual nature of the impact of problematic short video use.

## Conclusions

Extant research has suggested that problematic short video use is associated with a series of physical and mental health issues. In the current era of the prevalence of short videos, the potential relationships between this problematic behavior and suicidal ideation and self-injurious behaviors has yet to undergo rigorous empirical examination. We believe that problematic short video use has a dualistic impact. On one hand, it may temporarily alleviate stress and provide a brief respite from psychological distress or academic pressures. On the other hand, it can also diminish an individual's self-control, leading to an obsession with the virtual world and thus resulting in psychological issues, which, in extreme cases, may culminate in suicidal behaviors. Utilizing a questionnaire-based methodology, we studied freshman and sophomore students in mainland Chinese universities, collecting and analyzing relevant data during their campus’s closed-off management. The outcomes of our research are as follows:

First, the current study suggested that problematic short video use had a dual impact on SI and SIBs. Second, sleep disturbance and depression play a significant mediating role in the relationships between such problematic behavior and SI and SIBs. Our research findings have further fleshed out the mechanisms underlying the impact of social media/internet addiction on psychological issues such as depression, as well as extreme outcomes, including suicide. Besides, our findings increase understanding of the dual effect of problematic short video use on SI and SIBs and call for attention to the true extent of the influence of such problematic behavior on these complex psychological conditions. Moreover, given that sleep disturbance and depression are important mediators in their relationships, intervention programs should target the depressive symptoms that are associated with sleep problems to reduce the escalation of suicidality in adolescence.

Future research can further validate the robustness and generalizability of the findings presented in this study by conducting multi-time point investigations, incorporating a more diverse range of participants (e.g., expanding the age groups represented, comparing different cultural contexts across regions), and incorporating moderator variables to analyze how the mechanisms under investigation vary under various conditions.

### Supplementary Information

Supplementary file 1. 

## Data Availability

No datasets were generated or analysed during the current study.
