# Supplementary material for: The association between problematic short video use and suicidal ideation and self-injurious behaviors: the mediating roles of sleep disturbance and depression
Source: BMC Public Health. 2024 Jun 25;24:1689. doi: 10.1186/s12889-024-19191-5 (PMC11197212; doi:10.1186/s12889-024-19191-5)
Supplement: Supplementary file 1 [file 12889_2024_19191_MOESM1_ESM.docx]

**Table S1**

Model-based effect sizes for the observed correlations (N = 1, 009)

| Relationship | |  | *β* | Effect Size (*r*) | 95% CI |
| --- | --- | --- | --- | --- | --- |
| Var.1 | Var.2 |  |  |  |  |
| PSVU | D |  | .20 | .70 | [.14, .26] |
| SD | D |  | .65 | 1.15 | [.60, .69] |
| PSVU | SD |  | .19 | .69 | [.11, .26] |
| PSVU | SI |  | -.16 | -.16 | [-.22, -.09] |
| SD | SI |  | .001 | .50 | [-.09, .09] |
| D | SI |  | .66 | 1.16 | [.55, .76] |
| PSVU | SA |  | -.09 | -.09 | [-.16, -.03] |
| SD | SA |  | -.04 | -.04 | [-.13, .04] |
| D | SA |  | .54 | 1.04 | [.44, .64] |
| PSVU | NSSI |  | -.05 | -.05 | [-.11, .01] |
| SD | NSSI |  | .05 | .55 | [-.03, .12] |
| D | NSSI |  | .49 | .99 | [.40, .58] |

*Note*. PSVU = Problematic short video use; SD = Sleep disturbance; D = Depression; SI = Suicidal ideation; SA= Suicide attempts; NSSI = Non-suicidal self-injury.

*r* = *β +* 0.5*λ* (*λ* is an indicator variable that equals 1when *β* is nonnegative and 0 when *β* is negative)

**Table S2**

Mediating effects with covariates for problematic short video use on suicidal ideation and self-injurious behaviors (N = 1,009)

| Total, direct and indirect paths | *β* | 95% CI | ∣a * b / c’∣ (%) |
| --- | --- | --- | --- |
| Total |  |  |  |
| PSVU → SI | .05 | [-.02, .12] |  |
| PSVU → SA | .07 | [.002, .13] |  |
| PSVU → NSSI | .12 | [.05, .18] |  |
| Direct |  |  |  |
| PSVU → SI | -.16 | [-.22, -.09] |  |
| PSVU → SA | -.09 | [-.16, -.03] |  |
| PSVU → NSSI | -.05 | [-.11 .01] |  |
| Indirect |  |  |  |
| PSVU → SD → SI | .00 | [-.02, .02] |  |
| PSVU → SD → SA | -.01 | [-.03, .01] |  |
| PSVU → SD → NSSI | .01 | [-.01, .02] |  |
| PSVU → D → SI | .13 | [.09, .18] | 81.88% |
| PSVU → D → SA | .11 | [.08, .14] | 114.89% |
| PSVU → D → NSSI | .10 | [.07, .13] | 197.96% |
| PSVU→ SD → D → SI | .08 | [.04, .12] | 49.38% |
| PSVU→ SD → D → SA | .06 | [.04, .10] | 68.09% |
| PSVU→ SD → D → NSSI | .06 | [.03, .09] | 118.37% |

*Note*. PSVU = Problematic short video use; SD = Sleep disturbance; D = Depression; SI = Suicidal ideation; SA= Suicide attempts; NSSI = Non-suicidal self-injury.

**Table S3.**

Regression Models (N = 1, 009)

|  | **SI** | | | | | **SA** | | | | | **NSSI** | | | | | **SD** | **D** |
| --- | --- | --- | --- | --- | --- | --- | --- | --- | --- | --- | --- | --- | --- | --- | --- | --- | --- |
|  | **M11** | **M12** | **M13** | **M14** | **M15** | **M21** | **M22** | **M23** | **M24** | **M25** | **M31** | **M32** | **M33** | **M34** | **M35** | **M4** | **M5** |
| SEX | –.075^*^ | –.068^*^ | –.063^*^ | –.069^*^ | –.068^*^ | .043 | .046 | .051 | .044 | .046 | .042 | .045 | .052 | .046 | .047 | –.017 | .002 |
|  | (.032) | (.032) | (.030) | (.028) | (.027) | (.033) | (.033) | (.031) | (.028) | (.028) | (.033) | (.033) | (.032) | (.029) | (.029) | (.033) | (.032) |
| GRADE | –.179^***^ | –.179^***^ | –.153^***^ | –.126^***^ | –.123^***^ | –.113^***^ | –.113^***^ | –.084^**^ | –.053 | –.050 | –.116^***^ | –.105^**^ | –.092^**^ | –.066^*^ | –.063^*^ | –.086^**^ | –.107^***^ |
|  | (.031) | (.031) | (.030) | (.027) | (.027) | (.032) | (.032) | (.031) | (.027) | (.027) | (.032) | (.032) | (.031) | (.029) | (.029) | (.032) | (.031) |
| AGE | .218^***^ | .202^***^ | .191^***^ | .172^***^ | .172^***^ | .076^*^ | .069^*^ | .056 | .036 | .035 | .104^**^ | .086^*^ | .083^*^ | .066^*^ | .065^*^ | .039 | .060 |
|  | (.032) | (.033) | (.031) | (.028) | (.028) | (.034) | (.034) | (.032) | (.029) | (.029) | (.034) | (.034) | (.033) | (.030) | (.030) | (.034) | (.032) |
| PSVU |  | .101^**^ | .056 | –.040 | –.041 |  | .042 | –.008 | –.118^***^ | –.119^***^ |  | .075^*^ | .019 | –.071^*^ | –.073^*^ | .153^***^ | .285^***^ |
|  |  | (.031) | (.030) | (.028) | (.028) |  | (.032) | (.030) | (.028) | (.028) |  | (.032) | (.031) | (.030) | (.030) | (.032) | (.031) |
| SD |  |  | .294^***^ |  | .086^**^ |  |  | .331^***^ |  | .094^**^ |  |  | .279^***^ |  | .083^*^ |  |  |
|  |  |  | (.029) |  | (.030) |  |  | (.030) |  | (.030) |  |  | (.031) |  | (.032) |  |  |
| D |  |  |  | .494^***^ | .452^***^ |  |  |  | .562^***^ | .516^***^ |  |  |  | .469^***^ | .428^***^ |  |  |
|  |  |  |  | (.028) | (.031) |  |  |  | (.028) | (.031) |  |  |  | (.029) | (.033) |  |  |
| *R*^2^ | .079 | .089 | .172 | .309 | .315 | .014 | .016 | .122 | .302 | .308 | .018 | .021 | .097 | .220 | .225 | .034 | .097 |
| Adj. *R*^2^ | .076 | .085 | .168 | .306 | .311 | .011 | .012 | .118 | .298 | .304 | .015 | .017 | .093 | .216 | .221 | .030 | .094 |
| Num. obs. | 1009 | 1009 | 1009 | 1009 | 1009 | 1009 | 1009 | 1009 | 1009 | 1009 | 1009 | 1009 | 1009 | 1009 | 1009 | 1009 | 1009 |

*Note*. Standardized regression coefficients are displayed, with standard errors in parentheses. PSVU = Problematic short video use; SD = Sleep disturbance; D = Depression; SI = Suicidal ideation; SA= Suicide attempts; NSSI = Non-suicidal self-injury.

* *p* < .05. ** *p* < .01. *** *p* < .001.

**Table S4.**

Regression Models without outliers, influential points, and high leverage point (N = 989)

|  | **SI** | | | | | **SA** | | | | | **NSSI** | | | | | **SD** | **D** |
| --- | --- | --- | --- | --- | --- | --- | --- | --- | --- | --- | --- | --- | --- | --- | --- | --- | --- |
|  | **M11** | **M12** | **M13** | **M14** | **M15** | **M21** | **M22** | **M23** | **M24** | **M25** | **M31** | **M32** | **M33** | **M34** | **M35** | **M4** | **M5** |
| SEX | –.072^*^ | –.063 | –.054 | –.066^*^ | –.063^*^ | .044 | .047 | .057 | .045 | .047 | .041 | .047 | .055 | .045 | .047 | –.033 | .005 |
|  | (.032) | (.032) | (.031) | (.029) | (.029) | (.033) | (.033) | (.032) | (.029) | (.029) | (.033) | (.033) | (.032) | (.030) | (.030) | (.033) | (.032) |
| GRADE | –.152^***^ | –.153^***^ | –.139^***^ | –.109^***^ | –.109^***^ | –.083^*^ | –.083^*^ | –.069^*^ | –.034 | –.034 | –.089^**^ | –.089^**^ | –.076^*^ | –.049 | –.049 | –.053 | –.092^**^ |
|  | (.032) | (.032) | (.031) | (.028) | (.028) | (.033) | (.033) | (.032) | (.028) | (.028) | (.033) | (.033) | (.032) | (.030) | (.030) | (.033) | (.031) |
| AGE | .198^***^ | .183^***^ | .177^***^ | .156^***^ | .157^***^ | .047 | .041 | .035 | .011 | .011 | .088^*^ | .077^*^ | .072^*^ | .052 | .053 | .021 | .056 |
|  | (.033) | (.033) | (.032) | (.030) | (.029) | (.034) | (.035) | (.033) | (.030) | (.030) | (.034) | (.034) | (.033) | (.031) | (.031) | (.034) | (.033) |
| PSVU |  | .109^***^ | .067^*^ | –.030 | –.031 |  | .042 | –.001 | –.116^***^ | –.117^***^ |  | .082^*^ | .045 | –.045 | –.046 | .154^***^ | .294^***^ |
|  |  | (.031) | (.030) | (.029) | (.029) |  | (.032) | (.031) | (.029) | (.029) |  | (.032) | (.032) | (.031) | (.031) | (.032) | (.031) |
| SD |  |  | .273^***^ |  | .081^**^ |  |  | .279^***^ |  | .053 |  |  | .243^***^ |  | .065^*^ |  |  |
|  |  |  | (.030) |  | (.031) |  |  | (.031) |  | (.031) |  |  | (.031) |  | (.033) |  |  |
| D |  |  |  | .473^***^ | .434^***^ |  |  |  | .538^***^ | .513^***^ |  |  |  | .433^***^ | .402^***^ |  |  |
|  |  |  |  | (.029) | (.032) |  |  |  | (.029) | (.032) |  |  |  | (.030) | (.034) |  |  |
| *R*^2^ | .063 | .074 | .147 | .276 | .281 | .008 | .009 | .085 | .270 | .272 | .011 | .018 | .075 | .187 | .190 | .030 | .099 |
| Adj. *R*^2^ | .060 | .071 | .143 | .272 | .277 | .005 | .005 | .080 | .266 | .268 | .008 | .014 | .070 | .183 | .185 | .026 | .095 |
| Num. obs. | 989 | 989 | 989 | 989 | 989 | 989 | 989 | 989 | 989 | 989 | 989 | 989 | 989 | 989 | 989 | 989 | 989 |

*Note*. Standardized regression coefficients are displayed, with standard errors in parentheses. PSVU = Problematic short video use; SD = Sleep disturbance; D = Depression; SI = Suicidal ideation; SA= Suicide attempts; NSSI = Non-suicidal self-injury.

* *p* < .05. ** *p* < .01. *** *p* < .001.


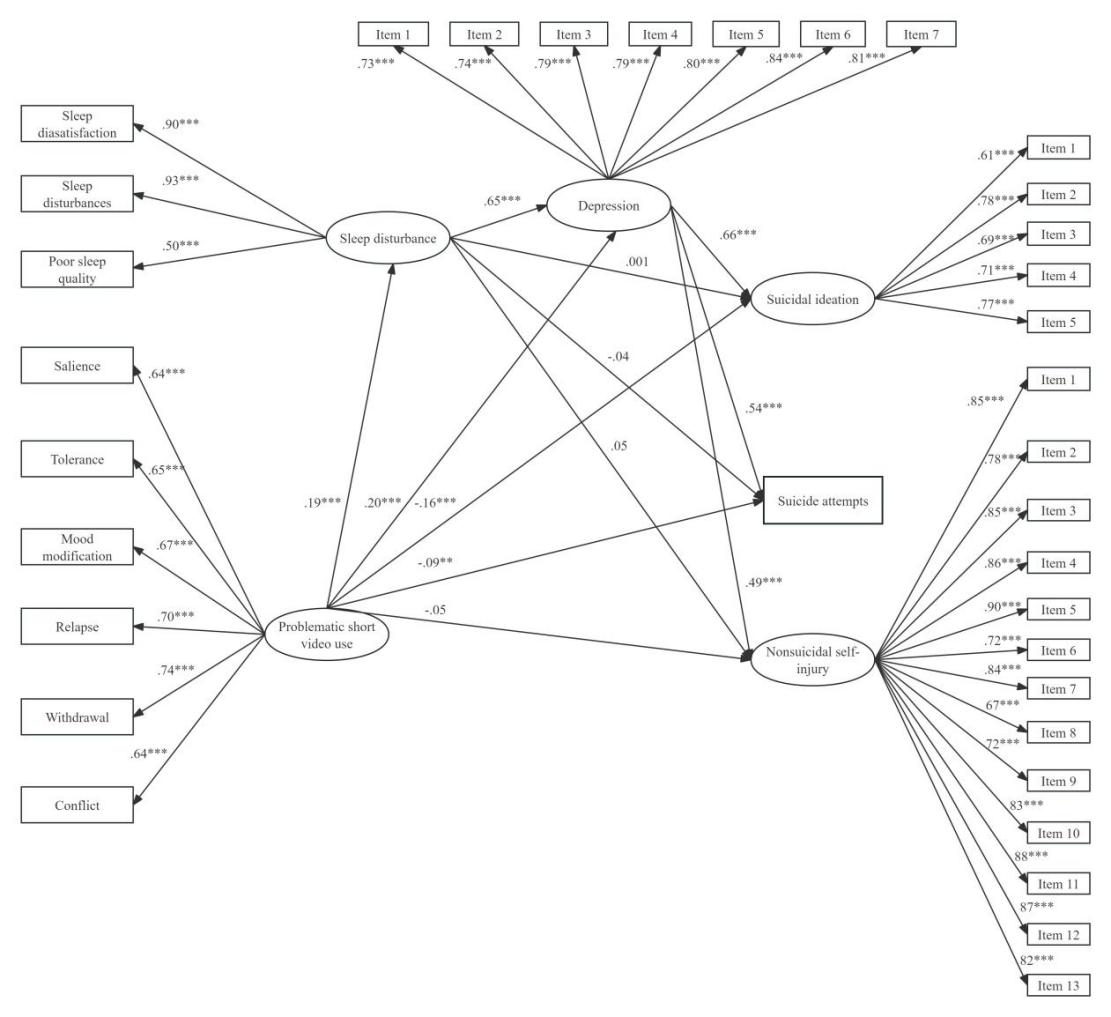


**Fig.S1.** Mediating effects of sleep disturbance and depression on the association between problematic short video use and suicidal ideation, attempts and NSSI (N = 1,009).

**p*<0.05; ***p*<0.01; ****p*<0.001.
